# Case Report: Acute Vision Loss in a Young Returning Traveler with Dengue Fever

**DOI:** 10.4269/ajtmh.20-0562

**Published:** 2020-09-08

**Authors:** Melina Heinemann, Eileen Bigdon, Luzia Veletzky, Sabine Jordan, Johannes Jochum, Volker Knospe, Stefan Schmiedel, Michael Ramharter

**Affiliations:** 1 Division of Infectious Diseases, I. Department of Medicine, University Medical Center Hamburg-Eppendorf, Hamburg, Germany;; 2 Department of Tropical Medicine, Bernhard Nocht Institute for Tropical Medicine, Hamburg, Germany;; 3 Department of Ophthalmology, University Medical Center Hamburg-Eppendorf, Hamburg, Germany;; 4 I. Department of Medicine, University Medical Center Hamburg-Eppendorf, Hamburg, Germany

## Abstract

Ocular complications are rare in patients with dengue fever, but may cause permanent loss of vision. We present the case of a 29-year-old German woman who developed severe acute vision loss because of dengue-associated maculopathy after traveling to Vietnam and Cambodia. Initially, the optical coherence tomography showed detachment of the retinal pigment epithelium, a central shift in the retinal pigmentation and intraretinal cysts. The patient was hospitalized and treated with a short course of intravenous prednisolone. Vision improved, and the patient showed full recovery at 9 months after the onset. This case highlights the importance of awareness and adequate management for ocular involvement in patients with dengue fever, including travelers.

## INTRODUCTION

Dengue is the most common arboviral disease worldwide, with an estimated global disease burden of 390 million infections annually, of which 100 million are symptomatic.^1^ The disease burden has increased greatly over the past decades, with Southeast Asia having the highest dengue incidence and mortality.^2^ Dengue is transmitted by *Aedes* mosquitos and has an incubation period of 5–10 days.^3^ Although most infections are asymptomatic, about 5% of patients develop severe, life-threating complications.^4^ Typical symptoms of dengue virus infection include fever, headache, retro-orbital pain, arthralgia, myalgia, nausea, vomiting, and rash. Despite the fact that ocular involvement is relatively uncommon, it is an increasingly recognized complication as it may lead to permanent visual impairment.^4,5^ Here, we present a case of severe vision loss due to acute dengue infection.

## CASE PRESENTATION

A 29-year-old Caucasian female patient presented at our outpatient clinic for tropical medicine, 4 days after returning from a 20-day vacation in Vietnam and Cambodia. She reported fever with body temperatures up to 39.1°C, headache, myalgia, sweating, and chills since 2 days. Furthermore, she suffered from diarrhea and vomiting, which started 7 days prior. Although the vomiting had disappeared, the patient still suffered from diarrhea at the time of presentation. She reported no previous comorbidities, except for mild bilateral myopia, and no medication intake. At admission, her white blood cell count was 2.6 × 10^9^/L (reference 4.4–11.3 × 10^9^/L), hematocrit 0.465 L/L (reference 0.35–0.45 L/L), platelets 158 × 10^9^/L (reference 150–400 × 10^9^/L), alanine transaminase 31 U/L (reference 10–35 U/L), and aspartate transaminase 39 U/L (reference 10–35 U/L). The diagnosis of dengue fever was confirmed by a positive nonstructural protein 1 antigen test and positive polymerase chain reaction. Serum anti-dengue virus IgM and IgG were initially negative, but seroconversion with detectable IgM and IgG in immunofluorescence test was observed after 1 month. Thick blood smear did not show any parasites, and the serology and polymerase chain reaction were negative for chikungunya virus. Because the patient did not show warning signs according to the 2009 WHO classification system,^6^ she was seen daily at our outpatient department. However, on the sixth day of fever, she experienced sudden onset of blurred vision as well as paresthesia on hands and feet, and she immediately presented at the emergency department of our hospital. This coincided with the nadir of her platelet count at 88 × 10^9^/L. The physical examination was normal, except for petechia at the hard palate.

**Figure 1.**
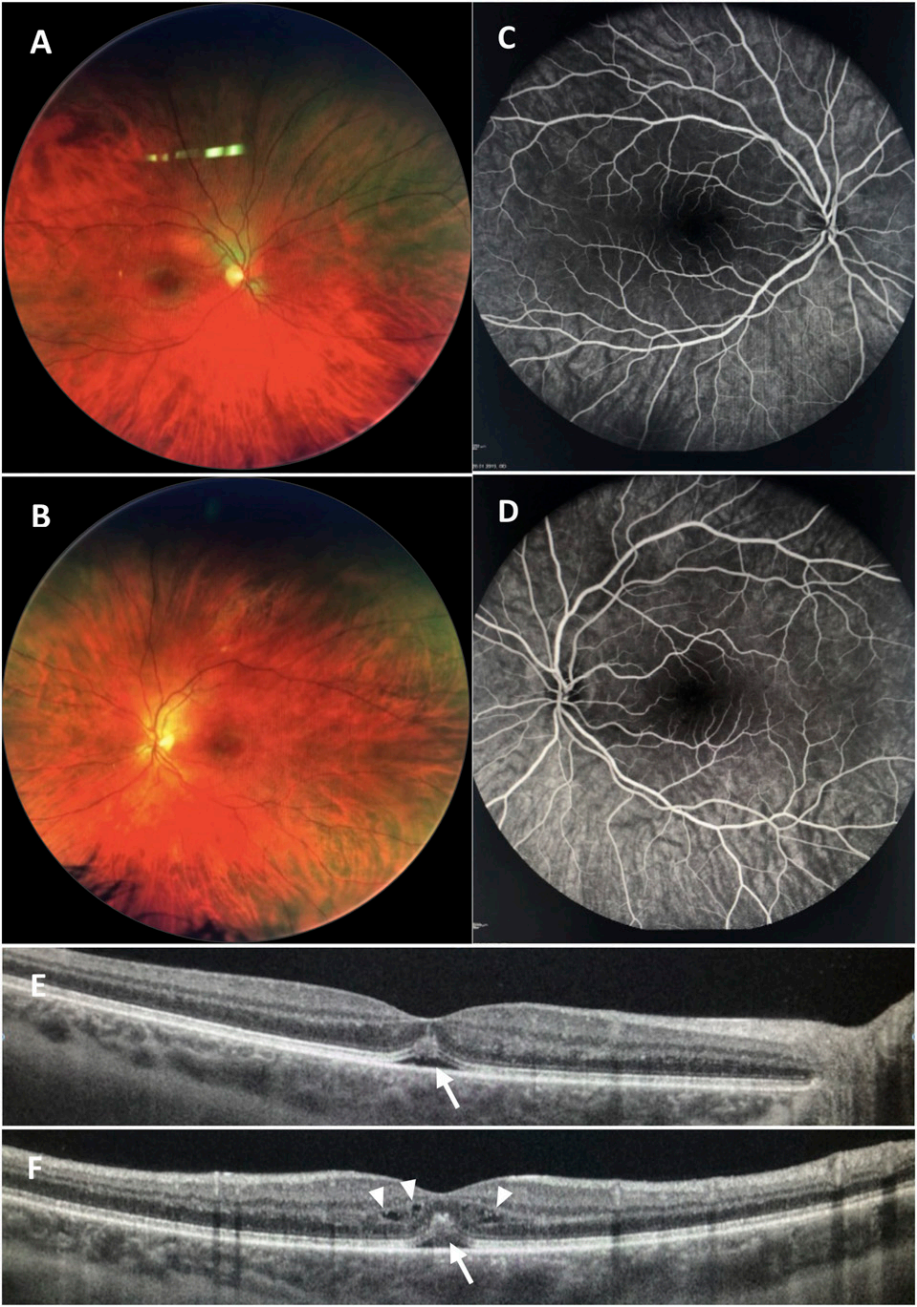
Multimodal imaging at the onset of visual loss. Normal fundus photography of the (**A**) right and (**B**) left eyes and normal fluorescein angiogram with no leakage on the (**C**) right and (**D**) left eyes. Optical coherence tomography showing detachment of the retinal pigment epithelium and subretinal fluid (arrow), central shift in the retinal pigmentation on the (**E**) right and (**F**) left eyes; intraretinal cysts (arrowheads) are more pronounced on the left eye. This figure appears in color at www.ajtmh.org.

At this time, the patient was hardly able to read because of vision impairment. Initially, the vision was finger counting on the left (oculus sinister) and 0.4 on the right eye (oculus dexter). Pupils were of equal size and reactive to light, with no relative afferent pupillary defect. Cerebral magnetic resonance imaging was normal. The anterior segment examination was unremarkable. The macula showed no reflex, the eyes showed no sign of vitritis, and the papilla was physiological (Figure 1A and B). The intraocular pressure was 15 mmHg in oculus dexter and 13 mmHg in oculus sinister (reference 10–21 mmHg). The fluorescence angiography presented no sign of leakage (Figure 1C and D). The optical coherence tomography (OCT) showed a maculopathy with a detachment of the retinal pigment epithelium, a central shift in the retinal pigmentation and fine intraretinal cysts in oculus dexter (Figure 1E). Oculus sinister showed similar pathologies with more intraretinal fluid (Figure 1F). The visual evoked potentials showed right/left similar patterns with prolonged latencies, oculus sinister > oculus dexter. The vision field testing showed unspecific changes, oculus sinister > oculus dexter.

**Figure 2.**
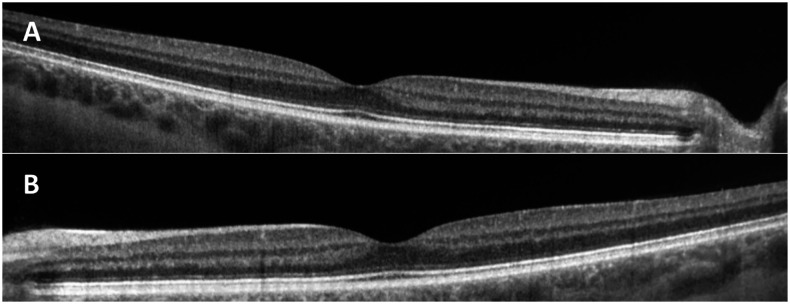
Optical coherence tomography of the (**A**) right and (**B**) left eyes showing regular neuroretinal layer without intraretinal fluid after more than 9 months.

The patient was hospitalized, and treatment with a short course of high-dose intravenous prednisolone of 250 mg/day was initiated. Reported steroid treatment duration varies from 2 weeks to 5 months.^7–9^ In the present case, prednisolone treatment was discontinued already after 2 days because of subjective improvement of vision as well as improvement in ophthalmological follow-up examination. Ten days later, the vision had improved to 0.1 in oculus sinister and 0.5 in oculus dexter. The OCT showed a discontinuation of the ellipsoid zone. More than 9 months after the onset, the patient had a full recovery, with a vision of 1.0 in both eyes and also a recovery of the visual field testing. The OCT showed a regular morphology (Figure 2A and B).

## DISCUSSION

Dengue-associated ocular inflammation is an increasingly recognized and reported ophthalmic disease often involving the posterior segment, with maculopathy being reported more often than other ocular manifestations.^4,10,11^ Maculopathy is defined as any pathological condition of the macula, which is the center of the retina and crucial for highly sensitive, accurate vision.^4^ Optical coherence tomography is an important diagnostic tool for characterization, monitoring, and prognostication of the visual defect in dengue maculopathy.^8,10,11^ Other useful diagnostic tools are visual field testing, fundus fluorescein, and indocyanine green angiography.^8,11^ The prevalence of dengue maculopathy was as high as 10% among seropositive patients in a study including 197 participants hospitalized for dengue fever in Singapore.^12^ However, there are only very few cases of dengue maculopathy reported in travelers. In the present case, other reasons for maculopathy cannot be excluded, but are very unlikely considering the results of the conducted investigations.

Ocular symptoms of dengue maculopathy can manifest unilaterally or bilaterally. The mean interval between onset of fever and onset of symptoms is 7 (range 0–30) days.^8,10,12^ Dengue-related ocular symptoms often present at the nadir of thrombocytopenia,^4,5^ as in the present case. Blurred vision is the most common visual complaint in dengue maculopathy^8,10^ and was the main visual defect described here. However, scotoma, micropsia, near vision disturbance, and floaters also occur in patients with dengue maculopathy.^8,10,11^

In patients with thrombocytopenia associated with dengue, venous occlusion with scattered blots and flame hemorrhages associated with perifoveal telangiectasia and Roth spots is common.^13^ Optic disc or diffuse retinal edema, retinal vasculitis, intermediate uveitis, anterior uveitis, vitreous cells, subconjunctival hemorrhages, or inflammatory optic neuropathy may also occur in patients with dengue fever.^4,8,11,13,14^ In severe cases, cotton wool spots can be present as a sign of occlusive vascular involvement.^13^ In a study including 41 patients with dengue maculopathy from Singapore, three patterns were proposed based on the predominant appearance on OCT imaging: type 1 with diffuse retinal thickening around the fovea, type 2 with cystoid macular edema characterized by intraretinal cysts disrupting the photoreceptor layers, and type 3 with foveolitis characterized by thickening and reflectivity at the subfoveal outer retina. Whereas type 1 was most common and had the best prognosis, type 3 had the worst prognosis with all patients noticing scotoma after 2 years of follow-up, even after clinical and anatomic structural resolution.^10^ According to this classification, in the present case, type 1 was observed in oculus dexter and type 2 was observed in oculus sinister associated with a more severe visual impairment at initial presentation. However, the vision was normalized in both eyes at the last follow-up.

The pathogenesis of dengue maculopathy is still unknown. An immune-mediated mechanism has been postulated.^8,12^ In patients with more severe ocular impairment, topical or systemic steroid treatment may benefit prognosis, with most patients achieving reasonable improvement of vision.^5,8^
